# On Contagion

**Published:** 1825-04

**Authors:** Charles Larkin


THE LONDON

### Medical and Physical Journal.

No. 4 OF VOL. LIII.] APRIL, 1825. [No. 314.

For many fortunate discoveries in medicine, and for the detection of numerous errors, the world is indebted to the rapid circulation of Monthly Journals; and there never existed any work, to which the Faculty, in Europe and America, were under deeper obligations, than to the Medical and Physical Journal of London, now forming a long, but an invaluable, series.—RUSH.

### ORIGINAL COMMUNICATIONS,

### SELECT OBSERVATIONS, &c.

ART. I.—*On Contagion.* By CHARLES LARKIN, Esq.

SINCE the late investigations, which were instituted by command of the House of Commons, in consequence of the impression produced by the works of that persevering and intrepid physician, Dr. MACLEAN, the subject of contagion has deservedly engaged much of the attention, both of the profession and of the legislature. Before introducing any alteration into the code of sanitary laws, the House of Commons wisely and prudently determined to derive whatever assistance they could, from the practical and theoretical knowledge of medical men. Unfortunately, however, the evidence of these gentlemen was so involved and contradictory, that, instead of illumining the subject, they only threw a thicker veil of darkness on that which was sufficiently obscure before; and, instead of resolving doubts, they only made those, who had entered upon the inquiry, and found it so difficult, nay impossible, whilst the road was entangled by such a mass of contradictions, to arrive at the truth,—seek relief in credulity from the pain of hesitation, and the anxiety which attends upon incertitude and indecision. Timid, distrustful of their own judgment, and hesitating to pronounce upon a question, concerning which the doctors disagreed, and upon which the learned casuists themselves had not made up their minds, the Committee hit upon the easiest, the readiest, and (in the meridian of St. Stephen's Chapel) the most approved method of deciding difficult and knotty points. Instead of giving a verdict upon the evidence, and entering into a full examination and comparison of the various facts elicited, they counted the number of physicians who advocated, and of those who opposed, the doctrine of contagion, and found that the majority was for the affirmative side of the question. "All the opinions of the medical men whom your Committee have examined, with the exception of two, are in favour of the received doctrine, that the plague is a disease communicable *by contact only*, and different in that respect from epidemic fever; nor do your Committee see any thing in the rest of the evidence which they have collected, which would induce them to dissent from that opinion." This mode of decision, which under the circumstances was full of wisdom and prudence, does not put a period to the discussion, but leaves the fact of the existence or non-existence of contagion in the same undetermined state in which it previously existed. The question, then, still remains open to fair, candid, and impartial investigation.

I say, fair, candid, and impartial investigation; for no inquiry, that is not entered upon in the spirit of fairness and candour, will conduct us to a right conclusion; and, most unfortunately for the cause of truth, upon this point the profession, to its eternal disgrace, is split into sects and factions, which are animated towards each other by the usual animosity and rancour which possesses a broken and divided community. Although this is a subject purely scientific, a mere matter of fact, it has been so long implicitly assumed and believed, that few will allow it to be questioned; and, like all old and reverend errors, it has called prejudice and passion to its support. This dogma, which in my judgment has no foundation in fact, but is based on misapprehension and inaccurate observation, is revered by those who cannot say any thing else in its defence than that it was believed by their fathers and grandfathers. With regard to things which are legitimate objects of taste and feeling, this circumstance strongly recommends them to our hearts and affections; but those things on which the happiness or misery of thousands depend, and which come more immediately under the cognisance of the intellect, ought not to be made matters of prejudice and passion. He that loves truth will not permit such sentiments to interfere with, or impede, the operations of his understanding; and will sedulously endeavour to wean his mind from all undue attachment to any set of doctrines and opinions. While he will avoid, on the one hand, the extremes of facility in the admission of new, and, on the other, of obstinacy in the retention of old, opinions, both of which are equally the characteristics of weak minds, he will preserve the safer middle path, and hold himself prepared to reject such as he may find to be false, and to embrace such as bear in their hand the passport of truth.

I do not, of course, intend to enter extensively into this subject. My object is to refute the contagionists, from their own concessions. Almost all my facts shall be taken from their works. Indeed, this Essay, and all the writings of the anti-contagionists, are almost works of supererogation; for no valuable work has yet been written to support the doctrine of contagion, which does not carry along with it its own refutation; so that, in placing their labours in the hands of the public, the intelligent reader will soon discern that "his bane and antidote are both before him."

I shall then proceed, without further preface, to explain the exact scope and drift of this Essay. This I cannot do better, than by attempting to explain a few of the various and opposite significations in which the words *contagion* and *infection* have been used. There is such a total want of precision in the meaning attached to them, that, unless I clear away verbal difficulties, and define the sense in which I shall use this word, contagion, we shall be involved in an interminable dispute. By this means I hope to reduce the question into a very narrow compass. It is the misfortune of professions, which endeavour to conceal their art and mystery from the profane vulgar in a peculiar phraseology, to exalt words to a greater importance in the mind of the student than things, and to give him a habit of repeating certain cabalistic sounds, on certain occasions, which communicate, neither to those who pronounce them nor to those who hear them, any very determinate meaning. Thus it is with these two words. They sound differently in every ear, and are used by no two writers in precisely the same meaning. With the earlier medical writers they were convertible terms. Some mean by them the producing cause, others the disease produced. Some have used the word *contagion* to signify those poisonous exhalations which are thrown off by diseased or putrifying animal or vegetable substances, which, diffusing themselves through the air, infect with disease the bodies of men and animals; and *infection* was used to signify the actual contamination of a healthy body by the morbific effluvia. Others have just reversed the matter, and mean by infection what these mean by contagion; and by contagion, what these mean by infection: by contagion implying the actual contamination, and by infection, the infectious matter. By others, again, infection is used, in general, to import that cause, whatever it may be, which engenders endemic or epidemic diseases; and contagion, the peculiar mode in which the subtle poison is admitted to the system,—that is, by close approximation to the sick, by contact, or by drawing in their breath, or by handling fomites. Others imply by contagion, not only the mode, but the matter of infection. Such are a few of the acceptations of these words, and such is a sample of the vagueness and ambiguity with which they have been employed by medical writers.

To the present hour, a distinct meaning has not been appropriated to them, and in all the above senses they are still used. To manifest the truth of what I assert, I shall contrast the manner in which they are employed, by two determined supporters of the contagion of plague, Drs. Granville and Johnson. The former of these gentlemen has very clearly distinguished infection from contagion, though it was not he who originally drew the line of separation. In the work to which I allude, he has very well illustrated what is meant by endemic, epidemic, infectious, and contagious diseases; and I may be here permitted to remark, that among epidemic, and not infectious or contagious, diseases, he has classed hospital, camp, and gaol fevers, typhus and various malignant fevers, croup, hooping-cough, and catarrh, and many others which have been, and still are, very generally regarded as contagious. I hail this approach to, as favourable to the truth of, the non-contagious doctrine, and as indicative that the disputes on this subject have led to more accurate remarks on the nature and character of diseases. I regard, as an augury favourable to its ultimate prevalence, this blotting out and unsparing erasure of so many diseases from the proscribed list. Formerly, almost all the diseases mentioned in the Nosology of Cullen were regarded as contagious. Just in proportion to the advance of knowledge, and the minuteness and accuracy of our observations, has the sphere of contagion been contracted; and its dominion is now shrunk to a few of the order Exanthemata.

Having then accurately distinguished endemic from epidemic diseases, Dr. Granville proceeds to observe—"If, during the prevalence of epidemic diseases, the deaths be numerous, and the burials carelessly conducted; if there be a great want of cleanliness in the persons attending the sick, and the sick themselves; if the effluvia arising from diseased bodies are not quickly carried away by ventilation, and the healthy be forced to mix with the sick, and breathe the atmosphere in which they linger, the disease then becomes infectious."

Attend, now, to the following answer which was given by Dr. J. Johnson, in his evidence before the Committee of the House of Commons, to the following question—"Do you believe the epidemic distempers and dysenteries of particular climates are contagious, under any circumstances?"—"I never knew of simple dysentery being contagious, but epidemic fevers may sometimes become so. I consider that *all fevers*, whether originally contagious or not, may become so, by the patients being too much crowded, by want of cleanliness, or by want of ventilation." Such are the contradictions which still perplex this subject.

To all who will give the slightest attention to this subject, I think it must be obvious that we ought to make a very clear and broad distinction between infection and contagion: the one operating at great and sensible distances; the other (whose existence I altogether deny), as its name implies, acting at insensible distances, or, at least, requiring a very near approach.

I do not concur with Dr. Granville in restricting the former term to the corruption and tainture of a confined atmosphere by distempered bodies; but I enlarge its signification, and make it embrace every thing and circumstance that has a tendency to vitiate the atmosphere, whether confined or free,—such as marsh miasmata, or the putrefaction of animals or vegetables, in the open air; or the crowding of people, whether healthy or diseased, into narrow and unventilated apartments. It is very well known that healthy bodies will vitiate a confined atmosphere, as quickly as those labouring under disease, and that those who breathe it will be exposed to attacks of malignant fevers. Mr. Howell, and others who escaped from the black-hole of Calcutta, were seized with typhus fever. The same fact has been observed in numerous other instances. As instances of the morbific and unhealthy nature of the gases which are extricated from putrid bodies, I beg to refer to the last Number of the Medico-Chirurgical Review, in which a case is related of two men, who, in digging a grave, happened to hit upon a spot in which another had been buried three months before. The instant the spade went through the lid of the coffin, a most dreadful effluvium issued forth, and the two men fell down nearly lifeless. They were dragged by their companions from the spot, and by their assistance were recovered. On the following day they were visited by the assistant surgeon of an Indiaman, who found them labouring under a severe attack of fever; acute head-ache, dimness of sight, giddiness, eyes of a peculiar muddy appearance, oppression about the præcordia, pain about the liver, diarrhœa, attended with symptoms of great debility, &c. On the fourth day petechiæ appeared; and, in one of the patients, a large bubo formed in his groin, and another in the axilla. All their symptoms exactly resembled those of plague. They both died. The fever, however, did not spread.—I may also just mention, that Forestus states that the putrefaction of a whale, which had been left on shore at Egmont, in North Holland, generated a very fatal and malignant fever. All these are instances of disease produced by *infection*; that is, proceeding from a cause which inquinates the atmosphere, and renders it prejudicial to the health of man. This, then, is Infection.

Contagion implies the exhalation of a specific miasm from the surface of the lungs and body of a person affected with a particular disease, which is capable of communicating the same disease to any other person who approaches or touches him, or inhales his breath, or handles his clothes. This is the doctrine which it is my intention to examine into the correctness of, in the following pages. Upon every principle of sound philosophy, I might ask, has the existence of this peculiar poisonous effluvium been proved?—has its existence been ascertained by experiment? If not, is it philosophical to assume that as actually existing, whose existence is only probable and conjectural? Ought not this proposition to stand in the form of a query, like Newton's conjecture about the existence of a subtle elastic ether? As far as I can discover, its existence has not been proved, but assumed for the sake of an hypothesis.—Again, with regard to the mode in which this poison is introduced into the system, the conjectures are as multifarious as the authors who have written on the subject. Some assert that actual contact is necessary to its imbibition; others, that a near approach is sufficient; a third set, that it is necessary to respire infectious breath; a fourth, and very numerous set, assert that the atmosphere around the infected person is tainted to the distance of many yards. By some we are told that the influence of this subtle emanation is immediate, extinguishing life with the rapidity of lightning; others, that some hours must elapse,—in some cases weeks, and in some, months. Such is an example of the congruence of the contagious philosophers, with regard to the properties and qualities of their subtle fluid!

Speaking of its virulence, Dr. Hodges tells us that "the deadly quality of a pestilence vastly exceeds either the arsenical minerals, the most poisonous animals, or the killing vegetables, (hemlock or the rattlesnake are nothing to it;) nay, the pestilence seems to be a composition of all the other poisons put together, as well as in its fatal efficacies to excel them. It is more active than lightning, and, in the twinkling of an eye, carries to a distance putrefaction, mortification, and death." In proof of this assertion, we have been amused with a variety of entertaining anecdotes. Boccacio tells us (and he states that he was an eye-witness of the fact,) that two hogs, finding in the streets the rags which had been thrown off a poor man, who died of the disease, after snuffling upon them, and tearing them with their teeth, these interesting animals fell into convulsions, and died in less than an hour! Dr. Mead tells us, that, in the year 1726, an English ship took in goods at Grand Cairo, whilst the plague was raging there, and carried them to Alexandria. Upon opening one of these bales in a field, two Turks employed in the work were immediately killed; and some birds, which happened to fly over the place, dropped down dead! *Credat judæus!* These are the *facts* upon which the doctrine of contagion is built! For my part, I do not think these marvellous tales form the very best foundation for a rational theory,—at least, if their works are addressed to men in the strength and vigour of their understanding. In weak minds, they may excite alarm and terror; but those in whom these feelings are not quite so easily excited, will only smile incredulously at them. Dr. Granville also gives us a story of a priest, who, upon touching an infected altar-cloth, was immediately seized with the plague, and in three hours died, with buboes in his arm-pits and groins. But I forget myself; it is only obstinacy and ignorance that can long remain sceptical upon these points; and I have to retract a hasty assertion that I made a short while ago, that the existence of this poisonous aura had not been ascertained by experiment. Not only was its existence ascertained, but it was actually subjected to analysis, during the last plague of London. The breath of a pestilent person was received upon a piece of glass, and was found to consist of an innumerable multitude of strange, monstrous, and frightful animals,—such as snakes, and serpents, and dragons, and devils, frightful to behold! The individual who gives us this account was a man of strong sense; and he observes, with imperturbable gravity, that he is rather inclined to suspect the truth of this narration, not so much from its improbability, as from the insufficiency of their instruments to effect such sublime and wonderful discoveries! The same author acquaints us with some other properties of this breath, not quite so terrific, to be sure, as those just enumerated, but sufficiently curious to deserve to be recorded for our instruction. It would instantly kill a small bird,—nay, even a cock and a hen; that, if it did not immediately kill the latter, it would cause them to be roupy; and, if the hen should lay any eggs at that time, they would all be rotten.

This is the best and most philosophical account which I have been able to collect from the works of the contagionists, of the nature, properties, and qualities, of this subtle and deleterious effluvium.

But, let the essence of contagion be what it will, I shall now examine the grounds upon which we are called upon to give our assent to the proposition, that some diseases are communicable by contact. It will be perceived, by what I have already said, that I intend to try the correctness of this doctrine, by referring to the history of the plague. I do this, because the accounts which we have of this fatal distemper are more full and correct than those which we have of any other disease reputed contagious; because its contagion is most dreaded, and, to restrain its progress, a code of sanitary laws has been framed, which, to speak gently of it, whatever publicly beneficial intention it was designed to fulfil, is grievously oppressive, and needlessly and uselessly harsh and cruel to individuals. I have chosen to attack the contagionist here, because I consider it as his seat of empire, his strong hold,—the citadel, to which, when all his outworks have been carried, he retires secure and fearless, and deems himself impregnable: because here, if contagion exist at all, it displays itself in its most terrific pomp and most tremendous grandeur;—because, when exerting its might in the shape of pestilence, it seems to collect all its energies, hangs blackening for a while, then precipitates itself, not only upon single towns, provinces, or kingdoms, but in one universal storm of woe, upon all nations, desolating empires, and half unpeopling the globe.

In the time of the Emperor Justinian, the Greek historian, Procopius, informs us, that the number of those who fell before the sword of this angel of destruction was μυριαδας μυριαδων μυριας; an expression, it has been observed, to which there are objections, both in grammar and arithmetic. A literal translation gives us a number too enormous to be credible,—ten thousand times ten thousand myriads; but, if we drop the μυριαδας, we shall have a number more within the range of probability. Our information will then be, that the ravenous maw of death was glutted with 100,000,000 of men. During three months, it is said, five, and at length ten, thousand persons died each day at Constantinople; many cities of the East were left vacant; and, in several districts of Italy, the harvest and vintage withered on the ground. During a calamitous period of fifty-two years, says an elegant historian, the subjects of Justinian were afflicted by the triple scourge of war, pestilence, and famine; and his reign was disgraced by a visible decrease of the human species, which has never been repaired, in some of the fairest countries of the globe.

On contemplating this enormous waste of human life, we can scarcely believe that an agent so feeble, and so easily capable of restriction, was competent to so mighty a destruction;—that a subtle poison, lurking in a bale of cotton for years, or in a feather bed, (Mead gives us an instance of its lying snug in a good feather bed for seven years,) suddenly breaking loose from its confinement, should sweep over and devastate the earth;—and that, after rioting awhile in the destruction of mankind, it should suddenly and capriciously cease to put forth its deadly energies.

The contagionist of the present day has greatly improved on the doctrines of his predecessors. He will admit no other cause of pestilence than contagion. The contagionist of old thought that it was the product of the united energy of various physical agents. Some thought it derived from stellar influence, from malignant aspects of the heavens, or evil conjunctions of planets; others thought it was shook from the "horrid hair" of comets; some thought it of supernatural origin, and acknowledge the το θειον of pestilence. Some thought it descended "in airs from heaven;" others, that it arose "in blasts from hell." Some ascribed its origin to the fall of meteors; others, to the projection of balls of fire into the heavens, from the bowels of the earth! Dr. Hodges thought it arose from the corruption of the nitrous spirit of the air. Sydenham, the illustrious friend of the more illustrious Locke, thought it depended upon some peculiar pestilential condition of the atmosphere; and Dr. Mead, a decided contagionist, and one of the most able and elegant of its advocates, considers a corrupted state of the air essential to its existence. Dr. Hodges and Dr. Mead, in proof of their opinion, observe, that blasts among trees, diseases among cattle, and fevers of unusual malignity, generally precede the birth of plague. In this opinion, there is a remarkable coincidence between the earliest writers and those learned and excellent men, and those who succeeded them, until "the flattering prospect of standing *unique* in the vast tide of general opinion, which rushes against them from every known part of the globe," tempted some of our contemporaries to broach the new opinion, that it is communicable by contact alone.

The earliest plague we read of in profane history, is attributed to the agency of intense heat. Apollo, incensed at the insult offered to his priest, afflicts the Grecian camp with pestilence; and Homer represents it as first attacking the mules and dogs.

> Οὐρῆας μεν πρῶτον ἐπωχελο, και κυνας ἀργους·
> Αὐταρ ἔπειτ᾽ αυλοισι βελος εχεπευκες ἀφιείς,
> Βαλλ αἰει δὲ πυραὶ νεκύων καίοντο θαμειάι.

*Iliad,* lib. 1.

The same observations are made, with the same clearness and distinctness, by Lucretius and Ovid. (See *Ovid,* lib. 7.)

> "Varius concinnat id aer.
> Proinde ubi se cœlum, quod nobis forte alienum 'st
> Commovet, atque aer inimicus serpere cœpit:
> Ut nebula, ac nubes paulatim repit, et omne
> Qua graditur, conturbat, et immutare coactat.
> Fit quoque, ut in nostrum cum venit denique cœlum
> Corrumpat, reddatque sui simile, atque alienum.
> Hæc igitur subito clades nova, pestilitasque,
> Aut in aquas cadit, aut fruges persidit in ipsas,
> Aut alios hominum pastus, pecudumque cibatus:
> Aut etiam suspensa manit vis aere in ipso;
> Et cum spiranteis mistas hinc ducimus auras
> Illa quoque in corpus pariter sorbere necesse'st."

*Lucret.* lib. 6.

It is remarkable that, in the whole of this passage, not one word is said about the importable nature of plague, a doctrine of which the ancients seem to have been entirely ignorant. These quotations I have thought it incumbent on me to introduce, in order to mark the great discrepancy between the good old opinion and the new-fangled hypothesis, "*that plague is communicable by contact only.*" Which is correct, remains for decision. This, however, these extracts distinctly prove, that, in the general belief of mankind, in these instances of great fatality, there is around and about us a cause producing disease and death, vastly exceeding in force, and having a wider circle of operation than, that which is included under the term contagion; to which they considered this but as subordinate and subsidiary,—a sort of gleaner in the field, following the heels of the great reaper. Whether it is consistent with the rules of just philosophy thus to multiply agents, when it is confessed that there is one in action equal to produce the effect, and completely ruling over and controlling it, is a question worthy of consideration. Indeed, I shrewdly suspect that it is the force of this objection that has induced the ultra contagionist of our day to discard the complexity of the old opinion, and give to his own a more philosophic air of simpleness. Are there not reasons to suppose that he has kept the worser half, and rejected the better moiety of the hypothesis? Of this we shall shortly be enabled to judge.

The statement now is, that pestilence is propagated by contact alone,—is not influenced by temperature,—obeys no change of season,—and is perfectly unaffected and unchecked by any condition of the atmosphere. Compare these assertions with these facts: In Ethiopia, it has been observed that the plague invades it whenever rains fall during the sultry heats of July and August. In this country, the vitiation of the air is occasioned by those immense swarms of locusts which infest it, the putrefaction of which is heightened by the excessive intemperance of the climate, in which violent rains fall, at one season of the year, for three or four months together. In enumerating its causes, I should have noticed, that numerous swarms of insects have frequently been observed to be the precursors of plague, producing it by tainting the air in their dissolution. So thoroughly were the Egyptians impressed with this fact, that Cicero informs us that they worshipped the bird Ibis, for the service it did them in devouring great number of winged serpents, which were wafted by the wind from the vast deserts of Lybia; thus rendering them innocuous, by preventing them from hurting by their bite when living, or their pestilent vapour when dead. The Arabian physicians, who lived near these countries, declare that pestilences are brought by unseasonable humidity, by heat, and want of winds. To show that its course may be checked by the state of the air, attend to the following fact:—At Smyrna, the plague constantly ceases about the 24th of June; dry and clear weather then succeeding to the unwholesome damps that annoy the country during the spring.

Whatever infected goods, or whatever infected persons, are landed during the winter-months, *plague never spreads*. Here, then, it is evident that it is not propagated by contact alone; that it does obey change of season, is affected by temperature, and checked by the condition of the atmosphere. Of this we shall have more proofs anon. But, before I proceed to accumulate facts against the contagionist, I must examine some of those which he brings forward as evidence of the truth of his opinion.

In the accounts which have been given us of the origin of every plague, there is so much variation and discrepancy, that it is a task of no ordinary labour to separate the pure truth from the shell and husk of falsehood, in which it lies involved. Every writer has his theory, and it is the business of each to make the *facts* (as they are called) square and tally with his particular notion. Hence a complexity and intricacy in their different relations, that defies all analysis; and as to their facts, they are either so marvellous, that, instead of compelling conviction, they strengthen incredulity, or they rest upon such slight and contemptible authority, as to form no base whatever to erect a rational belief upon. It was an observation well worthy of the acute and discriminating mind of Cullen, and one to which, by frequent repetition, he wished to give the currency of a proverb, that there were more false facts in circulation than false theories. This is a saying, whose truth every one, who has in the slightest degree trained his mind to habits of observation and reflection, must have had numerous opportunities of verifying. The remark is so valuable, and an ignorance of it must lead to so many errors in opinion and reasoning, that it was impossible it should escape the rapid and all-seeing eye of Bacon. Accordingly, we find a similar remark scattered through various parts of his works; and to the following quotation I beg the serious attention of my reader:—"Men of learning are too frequently led, from ignorance or credulity, to avail themselves of mere rumours or whispers of experience as confirmations, and sometimes as the very groundwork of their philosophy, ascribing to them the same authority as if they rested upon legitimate testimony. Like to a government which should regulate its measures, not by the official information of its accredited embassadors, but by the gossipings of newsmongers in the streets. Such, in truth, is the manner in which the interests of philosophy, as far as experience is concerned, have hitherto been administered. Nothing is to be found which has been duly investigated,—nothing which has been verified by a careful examination of proofs,—nothing which has been reduced to the standard of number, weight, or measure." What an admirable commentary on the manner in which the doctrine of contagion, and the importable character of plague, have been attempted to be proved!

In illustration of the correctness of this assertion, I shall quote the following sentence from the "Loimologia" of Dr. Hodges. Speaking of the plague of London, he says, "It was imported from Holland in packs of merchandise; and, if any one please to trace it further, he may be satisfied, by *common fame*, that it came thither from Turkey in bales of cotton." Truly, if we believe the contagionist, a few bales of cotton have entailed more mischief and destruction on the world, than ever were brought upon it by ambition or by avarice, by civil broils or by foreign wars. So we are very cavalierly told by the Doctor, that we are to be satisfied that it came from Turkey, because common fame said so; and the degree of fretfulness and irritation which the contagionist displays, if you refuse to be contented with these "gossipings of newsmongers in the streets," is very amusing. Why, I had thought that, ever since the days of the Roman poet, and before, the strumpet had been known for an arrant liar and notorious fabulist.

> Luce sedit custos aut summi culmine tecti

> Turribus aut altis, et magnas territat urbes.

> Tam ficti pravique tenax, quam nuntia veri.

That she is as multiplicious in her reports as ever, (for she is also characterised by the same observant poet as "multiplice sermone gaudens,") the following information respecting the same plague will show. We are told by the journalist of the plague of London, that the distemper had been violent at Amsterdam and Rotterdam in 1663-4. "To these places it was brought, some said from Italy, others from the Levant, among some goods brought home by their Turkey fleet; others said it was brought from Candia, others from Cyprus." This is a sample of the evidence on which the importable and contagious nature of pestilence is supported. Neither Dr. Hodges nor the journalist affect to trace any direct communication between the touched goods imported from Holland, and those first infected by the disease. In fact, no such communication existed; and the contagionist of that day had not the hardihood to tell us, that we must believe that communication did exist, though that communication was unsusceptible of proof.

My second illustration of the truth of Lord Verulam's remarks, I shall take from a work which has been said to place the contagious nature of plague beyond the reach of controversy. The work to which I allude is Sir A. B. Faulkner's Treatise on the Plague, designed to prove it contagious from *facts* collected during his residence at Malta. The author had every possible advantage,—having the disease under his eye, being officially employed, and in constant habit of communicating with those who had the direction of affairs. It is not my intention to enter into a minute criticism of the work: I only wish to select a few of his *facts*, to show the very unsatisfactory nature of the evidence which this writer, after condemning himself to seven years of hard labour, and with every possible advantage, was enabled to collect.

On the 28th of March, 1813, the San Nicolo, which our medical knight calls the "fons et origo malorum," applied to the Health Department for admission into port, previously using the precaution to notify her state, (two of the crew having died of plague on their road to Malta,) by hoisting a yellow flag, with a black ball in the centre; that being the signal to indicate the actual existence of plague on-board. She had brought a cargo of linen, flax, and leather, from Alexandria. Her application was acceded to, and she was received *into quarantine* in the Marsamuchet harbour, within about a cable's-length of several points of land, and of the city of Valetta. The surviving part of the crew were taken into the lazaretto, situated in a small island in the middle of the harbour. The captain and his servant sickened in a day or two after their admission in the lazar-house, and died with indisputable symptoms of the plague.

I may here be allowed to remark, that great emphasis has always been laid upon the arrival of some ship or goods from the Levant, or from some place having frequent communication with that suspected part of the world, some three, six, or eight weeks previous to the eruption of plague. That such an occurrence should happen to precede the commencement of a fatal epidemic, is not very extraordinary, if we reflect upon the constant intercourse that has subsisted, for a very long period of time, between the Levant and the different ports of Europe: indeed, it would be not only an extraordinary, but an almost impossible circumstance, when we consider that vessels are continually arriving, that such an occurrence should not antecede it. I cannot, therefore, be inclined to lay much stress upon an accident of this kind, until I find it coupled with other circumstances of a much stronger nature than any which it has yet fallen to my lot to be acquainted with. But suspicion and an ill name is attached to every thing coming from that part of the world; and, from the propensity which there is in the human mind to connect the idea of causation with that of antecedence, no evil can arise, after such an occurrence, without its being attributed to that source. In illustration of this propensity to draw hasty conclusions, Dr. Reid mentions that, upon a great number of miscarriages being accidentally succedent to the arrival of a white cow in a country village, where such a prodigy had never before been seen, the rustic philosophers, with a nose not a whit less sagacious than that of the contagionist, soon discovered in the white cow the "fons et origo malorum," and never allowed themselves rest until that ill-starred animal was expelled the vicinity.

The first character in which the Doctor wishes to appear, is that of a prophet; and, like most prophets, he comes to denounce suffering and woe. Filled with very natural apprehensions at the arrival of this plague-ship, with the ardour of another Laocoon, he deprecated the admission into port of a machine, which contained within it more evil than was included in the fatal horse of Troy. He addressed a letter to Lieutenant-general Oakes, in which he ineffectually urged him to adopt defensive measures against the terrible enemy that had appeared on the coast. The date of this letter is April 10. On the 16th of the same month, a girl was taken ill with a disorder, which perhaps was plague, though the fact is by no means clearly ascertained. Having stated these three circumstances, the Doctor asks, with the air of one who was about to be delivered of something that would flash conviction on every mind, whether, when we join together the circumstances of the presence of the San Nicolo, the awful warning of his letter, and the eruption of the plague a few days after it was written, the conclusion that the San Nicolo was the cause of the plague, be not "one of those consecutions (quoting the words of Hale,) which are so intimately and evidently connexed to, or found in, the premises, that the conclusion is obtained *quasi per saltum*, and without any thing of ratiocinative process, even as the eye sees its object immediately, and without any previous discourse?" For my part, I am obliged to confess that my mental vision is so blunt, that I can discover no connexion whatever between these premises and this conclusion; nor do I profess to entertain any great respect for the sect of philosophical *jumpers*, whose method of procedure has been very admirably described by a humorous and satirical poet:—

> "Where others toil with philosophic force,
> These nimble fellows take a shorter course,
> Fling at your head conviction in a lump,
> And gain remote conclusions at a jump."

Before I give my assent to the proposition, that these things stand to each other in the relation of cause and effect, they must be shown to me to be much more closely connected than by the accidental precedence of the one and subsequence of the other. As to the letter, to which much importance is attached, nothing could be more natural than that a firm believer in the orthodox doctrine should feel alarmed at the abrupt appearance of plague in his immediate neighbourhood, at a time when it was actually raging in different parts of the Levant. *Proximus ardet Ucalegon!* Ucalegon's house is actually on fire, and the Doctor would have us believe that there is something very wonderful and confirmatory of the truth of his creed, in his beginning to tremble for the safety of his own!

Three weeks elapse, from the arrival of the vessel, before the plague reveals itself in Valetta, and it alights first on a little girl, the daughter of Salvator Borg, a shoemaker. My reader will be naturally inclined to suppose that this illustrious cobbler lived somewhere in the vicinity of the plague-ship? No; in the very heart of the city. That he, or some portion of his family, had communicated with the infected crew? No; not with one. Had undoubtedly handled the infected goods, then? This we should, of course, suppose was completely proved,—so completely, as not to leave the least shade of doubt upon the mind. My readers shall judge. Dr. Faulkner himself shall speak:—"The manner in which the plague was introduced into Valetta from the San Nicolo, was *confidently stated in Malta* to have been by a piece of linen, part of her cargo, which was purchased by one Salvator Borg, a shoemaker." Here again, then, we have bold assertion, and the "gossipings of newsmongers in the streets," to establish the validity of a doctrine, which (to use a strong expression, applied by Dr. Granville to the doctrines of Brown,) is itself a plague. If, really, the Doctor expected to find readers who would be contented with proofs like this, he paid a compliment to their docility, which all, I hope, have had understanding sufficient not to deserve. How proud the anti-contagionist must feel, when he contrasts his arguments and facts with those which his adversaries oppose to them! Common rumour, and things *confidently stated* in the streets of Malta!

The reluctant acknowledgment is wrung from our author, that he wants facts derived from satisfactory authority, to establish fully an actual communication between the San Nicolo and the city. So mysterious and inscrutable was the manner in which the pestilence entered Valetta, that of this piece-of-linen story we have different readings. Dr. Granville tells us, that to this day the Maltese firmly believe that S. Borg, who was a shoemaker, purchased some linen to line shoes. From some one belonging to the vessel? No; from a Jew. Who had, by some underhand means or other, got it from the vessel? No; who had received it from Alexandria.

So little credence does Dr. Calvert place on those idle stories, that he rejects them totally, and recurs to the ancient doctrine that the air became infected, and carried disease and death into every quarter of the city: thus accounting for the plague of

Malta, in the same manner as Dr. Hodges accounted for the plague of London,—that the disease was communicated, not by actual contact and handling of infected goods of any description, but through the medium of the atmosphere? Is it necessary, then, to proceed further with this work? Is there not here a most lamentable gap? After using for several years every possible effort to amass facts and accumulate evidence, we have Sir A. B. Faulkner himself confessing *(habes confitentem reum,)* that he wants proofs to establish (and this forms the cardinal point of the case,) an actual communication between the "*fons et origo malorum*" and the first infected family in Valetta. The very first link of the chain is confessedly wanting. I ask then, with confidence, if idle rumour be a base sufficiently stable to erect so dreadfully important a doctrine as that of contagion upon? If it be not, then in the case of Malta there is not a shadow of evidence to prove that the disease which raged there in 1813 was imported from Alexandria.

The Doctor next proceeds to give eight instances of the disease, in which, according to him, it was extended in a direct line from one individual to another. The upshot of these instances is, that S. Borg himself, and the whole of his family, fell a prèy to the disease; and three others, who had either actually communicated, or who had held communication with those who had communicated with his family. His account is very meagre; and, though he tells us nothing more than just what he deems to be necessary to the end he has in view, there is nevertheless much that is open to animadversion. But I abstain from this, as I do not wish at present to enter into a minute criticism of his work, or draw out this Essay to an immoderate length. A document is produced to prove that, with the exception of these eight persons, no other individual was infected in any part of Malta. It is dated May 15th. It concludes in these words:—"No other case has occurred in the whole population which is deserving of remark." *Chi merita essere rimarcato.* Unless these words be an useless pleonasm, they mean that there were other cases. If there were not, why not unhesitatingly and unequivocally affirm that there were not? If there were, for what purpose are they concealed? We are next told that, after the 15th of May, the plague burst out in such a variety of directions, that no diligence of pursuit could follow up the direct line of contamination. Yet, in the teeth of this acknowledgment, and in utter contempt of all just rules of reasoning, it is affirmed, with all the confidence with which a positive and well established fact could be stated, that the disease was communicated in regular succession from one individual to the other. Because in eight cases (though the mode of contamination in the very first case is unknown,) an impression is attempted to be made, in an *ex parte* statement, that the disease was extended in a direct line from one person to another; therefore it was so extended in 4486. To my mind, this reasoning does not carry conviction. Again, is it likely,—is it probable, that the family of an obscure individual, which, the moment that it was known that plague had appeared in it, was held suspect by every person, and consequently avoided, should so completely carry pollution into every division of a large city, that, in one month after its first appearance in his family, it should burst out in so many directions, and in such a multitude of families, that to track the footsteps of contagion was impossible? Does not this look like a most violent assumption, forced upon us in order to bolster up an untenable supposition? It has neither the front or colour of a probable hypothesis. That it did so burst out, I have no doubt, because this is the custom with epidemics, to be lingering a while with a few families, then suddenly and at once to fall upon many. It is against the cause assigned that I argue, which I hold to be insufficient to produce the effect. I consider it produced by a cause that has a more extensive and general operation, and which is capable of acting simultaneously upon a large body of men.

If one concatenation of circumstances should fail to convince the heretical obstinacy of those who will not subscribe to the articles of his creed, our author, like an experienced tactician, has always a body of facts in reserve, which he pours in upon us in exhaustless succession, in hopes of producing an impression by a quick and rapid series of impulses, which no one was singly adequate to produce. He pursues the disease into the Augustan convent, which was situated in a peculiarly healthy, spacious, and airy part of Valetta. This convent stood near the upper part of one of the main streets, was elevated considerably above the level of the sea, and overlooked the whole town. Its neighbourhood was clean and open; its interior accommodations spacious; and its inmates remarkable for their respectability, decency, and good order. Into this asylum of virtue the disease was introduced by a servant. This individual, in disobedience of public orders, went, it is said, into a very contaminated part of the town, called the Manderaggio, and purchased infected clothes. Here again we are to be contented with mere assertion; no proof whatever is brought that htese clothes were infected. He was attacked with plague. One of the brotherhood, out of compassion, and a sense of religious duty, volunteered to attend, and placed himself and his patient in strict quarantine. Both fell victims to the disease; but no other individual under the same roof was ever assailed. This fact is given upon the testimony of a priest belonging to the convent.

Throughout the whole work, it is very remarkable how little the Doctor has to produce of his own. The eagerness and avidity with which the contagionist pounces upon a solitary accident of this kind, which seems to depose in his favour, completely exposes the poverty of his resources, and the labouring nature of his case. I do not, however, admit this to be an instance of contagion. But the case, even as related by the Doctor, speaks more against him than for him. Is the circumstance of its not being communicated to any other individual under the same roof, nothing? This is attributed to the strictness of the quarantine that was observed. But had this servant, before the complaint had manifested itself in him, not communicated with any other individual in the house? It is not affirmed that he did not. Neither are we told who became the attendant of this individual when he became sick, nor whether that attendant was at all infected with the disease. But, to weaken the effect this solitary accident may, by possibility, produce in some minds, I beg the attention of my readers to the following questions, put by the Committee of the House of Commons, and the answers which were given to them by Sir A. B. Faulkner:—"Who were the persons employed under you in the care of the sick?" "Orderlies, and such like."—"Did any one of them catch the plague?" "Not one."—"Were they necessarily in contact with the individuals who had the plague, and with their clothes and bedding?" "Necessarily."—"Had you the plague?" (The Doctor's answer was a very queer one:) "*I believe not; though I have some* DOUBT *respecting that.*" In opposition, then, to this solitary accident, I triumphantly place these numerous instances of individuals, who, from the author's own statement, though necessarily exposed to the utmost virulence of the contagion, in contact with the sick, and handling their clothes and bedding, did not catch the plague. But, as I do not at all wish to blink the question, I may here observe, that, though the death of this high-spirited individual, who volunteered his services to attend upon a man deserted by the rest of the community, is greatly to be regretted, yet he was, from the peculiarity of his circumstances, more exposed than any of his other brethren to the invasion of the disease, from anxiety, from fatigue, and from the depressing influence of terror, which it is difficult for a man, believing in this doctrine, not to feel when he finds himself shut up in the same room with a pestilent being.

The immunity of the convent is attributed to their seclusion, and their careful avoidance of all intercourse with the infected. This is the manner in which the contagionist views the question, giving all the credit and glory to his artificial regulations, nothing to the agency of nature. I think the fact (which has been observed in other plagues,) is capable of a very different explanation, which will restore to Nature what has been taken from her and given to Art. When the plague was last in England, while it was raging in Cambridge, the colleges were exempt from the distemper. In the plagues of Rome in 1656-7, the monasteries and nunneries were free from sickness. These places are the abodes of simplicity and virtue: in their peaceful halls, the shout of revelry or the brawl of intemperance is never heard; nor does the morning sun ever break in upon the late carousal. There men lead a tranquil and regular life: with hearts free from the corrosion of care, they calmly pursue the noiseless tenor of their way, neither soured by envy, galled by disappointment, nor stung by the thorns of a restless ambition. Abstinence gives to their constitution strength, to their bodies health, and cheerfulness to their minds. The same cause is in operation here, which, according to the remark of Aulus Gellius, enabled the divine Socrates, during the plague of Athens, to breathe untainted a tainted atmosphere; and with no other amulet around his neck than temperance and virtue to resist infection, and defy the inquination of the elements around him.

I shall not follow the Doctor through the barren and uninteresting waste of the account which he gives of the introduction of the disease into the casals, or inland towns and villages. In some of the casals, the mode of introduction is unknown, and in others it rests upon no better testimony than rumour, or the authority of a captain of the port,—a man to whom this doctrine of contagion is meat and drink. I cannot, certainly, but admire the pious simplicity with which the Doctor consulted this oracle; but I do not think he is quite the kind of oracle whose responses should be listened to with child-like docility and submissive reverence. Instead, then, of dissipating the attention of my reader over a vast extent of surface, I shall concentrate it upon the history of the disease in Bircarcara, Curmi, and Zebbug.

We are told by him that, if we look at the casals in reference to their respective distances from the common source of contamination, Valetta, and from the towns most infected in their neighbourhood, we shall find, just as we ought to expect during the prevalence of a contagious disease, that the degree of severity with which each suffered was in the ratio of its degree of communication with these sources of infection; or in the ratio compounded of its direct communication with and vicinity to Valetta, and the adjacent infected towns. Now, let us examine the truth of his doctrine by the very criterion which he places in our hands. Curmi lost 530 of its inhabitants, and is distant from the great nidus of infection only two miles, and is more exposed to communication with Valetta, lying upon the direct road. Zebbug is distant four miles from Valetta, and lost 675. Of all the casals, this was most frightfully ravaged; and why? Because, says the Doctor, it was situated on the high road of contagion. He means to say, I presume, that its infection was derived from a double source, Curmi and Valetta. With these two casals, which submitted to the discipline of the contagionist, compare Bircarcara. This casal is distant from Valetta two miles, from Curmi half a mile. Being, therefore, both nearer to Valetta, the great source of infection, and to Curmi, it was as completely upon the " high road" of contagion as Zebbug, which is distant four miles from the one, and two from the other. In addition to this, the greatest degree of insubordination prevailed; the town was very populous, and its inhabitants impatient, to the highest degree, of all restriction. They had no faith in the lines and barriers of the contagionist, and wisely refused to submit to his tyranny. Was the mortality, therefore, greater in Bircarcara than either at Curmi or at Zebbug? It ought to have been so; for we are told that the degree of severity with which each casal suffered " was in the ratio compounded of its communication with and vicinity to Valetta, and the adjacent infected towns." What, then, is the fact? In this populous town,—in this town in direct communication with and vicinity to Valetta and Curmi,—in this town, in which the greatest insubordination prevailed, which absolutely scorned all restriction and precaution, only 250 persons died: that is, less than one-half of the number of those who died at Curmi, and, within a fraction, only one-third of the number of those who died at Zebbug; which both submitted to the regulations of the contagionist, and the mortality of both of which give ample and abundant evidence of the prudence and wisdom of the discipline he recommends. If, then, there was either correctness in his reasoning, truth in his belief, or virtue in his regulations, which convert every man's home into a prison-house, instead of Zebbug, of all the casals, Barcarcara should have been most frightfully ravaged.

In order to show that the plague of Malta was totally independent of the atmosphere, and thus indirectly prove that it must have been imported, we are told that the plague did not occur there for 130 years. If it arose from atmospheric causes, it must have arisen at some period during that time, as the recurrence of every possible cycle of combination, in regard to the elements of atmospheric impurity, must have taken place. Therefore, the fact of its non-recurrence during that period proves that it was imported, and propagated by contagion alone. That mind must be strangely constituted, of a " curious organisation," that can discover any force in this argument. It is a downright assertion, and no argument. The thing must have occurred,—therefore it did occur. Estimating the argument at its highest value, it is but a *petitio principii.* The Platonic year, I should think, would embrace a much longer cycle than this: 130 years is but as a point in the great circle, a mere atom in the great bank and shoal, of time.

In the opinion of the contagionist, two conditions are indispensably requisite to the propagation of pestilence,—the existence of a specific virus, of the nature of which he professes himself ignorant; and a peculiar aptitude in individuals to receive and be affected by this "airy nothing," of which assumed aptitude he equally professes his ignorance. Mark now how the individual who assumes the necessity of the co-existence and co-operation of these two occult causes in order to the production of the disease, speaks of the hypothesis of a Sydenham, who supposed that a latent alteration of the air, some peculiar, indefinable, pestilential condition of the atmosphere, was necessary to the extension of plague:—"Did not so many writers of the present day insist upon the existence of this occult influence, I should not have considered it entitled to a serious notice, as the very mention of an occult cause shuts the door against all kind of reasoning." What is contagion, but an *occult* cause? What is this peculiar aptitude to receive the contagion, but an *occult* cause? Yet this very individual, whose whole book is but written for the support of an hypothesis, speaks with supreme contempt of the framers of hypotheses, and, with the pride of a Newton, is ready to exclaim, "Hypotheses non fingo!" Sir A. B. Faulkner does not consider the conjecture of a Sydenham, which comes recommended to us by the authority of physicians, philosophers, and poets, and by the universal testimony of mankind in all ages, worthy of serious notice! This is the manner in which, conjecturer himself, he rides over the conjectures of other men.

In reference to this subject, I may be indulged in the following observation:—It is not logical to argue, from the general healthiness of a climate, and general purity and salubriousness of the air, that the one will be perpetually healthy, and the other eternally pure and salubrious; or that, because our eudiometers, which are not sufficiently nice and delicate to inform us of every minute and peculiar change, indicate no alteration, none exists. In the scale of the chemist, the healthful mountain air, and the atmosphere of the foulest alley of St. Giles', have been found equal; but it does not, therefore, follow that they are both equally fit for all the purposes of life and health. In history we read of epidemics that, originating in some distant part of the globe, have thence progressively travelled into every country, following the course of the winds, and in a short space of time have swept over the earth, and involved in the same indiscriminate destruction the inhabitant of every climate. Differences may exist in that element in which we live, move, and have our being, important enough to destroy health, and extinguish the life of man, which may yet be far too subtle for instrumental detection. It is not wise to pronounce authoritatively upon a subject which is hidden from us in utter darkness. Many diseases, fatal to human existence, spring from causes mysterious and wonderful in their operation, acting with the utmost malignity on some, and passing others by unharmed, which are still among the arcana of nature. They lie concealed in some more interior recess, some holier and far more sacred penetrale in this great temple, than any into which the hand of investigation has yet led the adventurous and progressive mind of man. Whether they be scrutable or inscrutable, time alone, the greatest of discoverers, will make known.

In concluding this part of my paper, I may remark, that it is not at all accounted for why plague never broke out at any intervening point of time between the two last plagues, as it is confessed that the island was visited by plague-ships; and it is not shown that the vigilance of the officers of the Health Department was more relaxed in 1813 than at any intermediate portion of time. There are other circumstances incidentally mentioned in the work, which point to some other origin of the disease than contagion. We are told that the number of infected was increased by a high wind; and that the natives of colder climates, and especially the English, were unsusceptible of the complaint. If it be solely propagated by contact, and totally independent of all atmospheric influence, I cannot divine why a high wind should increase the number of the infected, or why it should pay more respect to the person of an Englishman than the body of a native. It is also deserving of record, that none of the men who navigated the San Nicсolo back were affected in their health: neither must it be forgotten that at this period (1813), and a year previous and a year after, the plague was raging with violence in different parts of the Levant.

[*End of the first Part.*]

